# Does Haplodiploidy Purge Inbreeding Depression in Rotifer Populations?

**DOI:** 10.1371/journal.pone.0008195

**Published:** 2009-12-07

**Authors:** Ana M. Tortajada, María José Carmona, Manuel Serra

**Affiliations:** Institut Cavanilles de Biodiversitat i Biologia Evolutiva, Universitat de València, València, Spain; University of Liverpool, United Kingdom

## Abstract

**Background:**

Inbreeding depression is an important evolutionary factor, particularly when new habitats are colonized by few individuals. Then, inbreeding depression by drift could favour the establishment of later immigrants because their hybrid offspring would enjoy higher fitness. Rotifers are the only major zooplanktonic group where information on inbreeding depression is still critically scarce, despite the fact that in cyclical parthenogenetic rotifers males are haploid and could purge deleterious recessive alleles, thereby decreasing inbreeding depression.

**Methodology/Principal Findings:**

We studied the effects of inbreeding in two populations of the cyclical parthenogenetic rotifer *Brachionus plicatilis*. For each population, we compared both the parental fertilization proportion and F1 fitness components from intraclonal (selfed) and interclonal (outcrossed) crosses. The parental fertilization proportion was similar for both types of crosses, suggesting that there is no mechanism to avoid selfing. In the F1 generation of both populations, we found evidence of inbreeding depression for the fitness components associated with asexual reproduction; whereas inbreeding depression was only found for one of the two sexual reproduction fitness components measured.

**Conclusions/Significance:**

Our results show that rotifers, like other major zooplanktonic groups, can be affected by inbreeding depression in different stages of their life cycle. These results suggest that haplodiploidy does not purge efficiently deleterious recessive alleles. The inbreeding depression detected here has important implications when a rotifer population is founded and intraclonal crossing is likely to occur. Thus, during the foundation of new populations inbreeding depression may provide opportunities for new immigrants, increasing gene flow between populations, and affecting genetic differentiation.

## Introduction

Inbreeding depression, or the decrease in fitness due to mating between relatives, is an important ecological and evolutionary phenomenon, which has been widely studied from multiple perspectives, such as population genetics, mating system evolution or conservation biology, and for a broad range or organisms [1], [2]. When a population is founded by a few individuals, inbreeding depression by drift may occur. The relationship between inbreeding depression and gene flow has emerged as a relevant topic to explore in the case of planktonic invertebrates inhabiting lakes and ponds (mainly cladocerans and rotifers), due to the population genetic structure detected for these organisms through molecular studies. Planktonic invertebrates are thought to have high passive dispersal abilities via resting stages [3]–[6]. However, strong differentiation in neutral genetic markers and in ecologically relevant traits has been reported [7]–[10], which suggests low levels of gene flow. De Meester *et al.* [11], extending the founding effects hypothesis of Boileau *et al*. [12], proposed the Monopolization Hypothesis to explain this paradox. According to this hypothesis, founding effects would persist due to (1) dilution, after population founding, of new immigrants in a large number of residents and (2) selection against immigrants arriving in a locally adapted population. Fast population growth from a few founders, persistent large population sizes, reinforced by large abundance of diapausing stages, and rapid local adaptation would make possible these effects.

**Figure 1:**
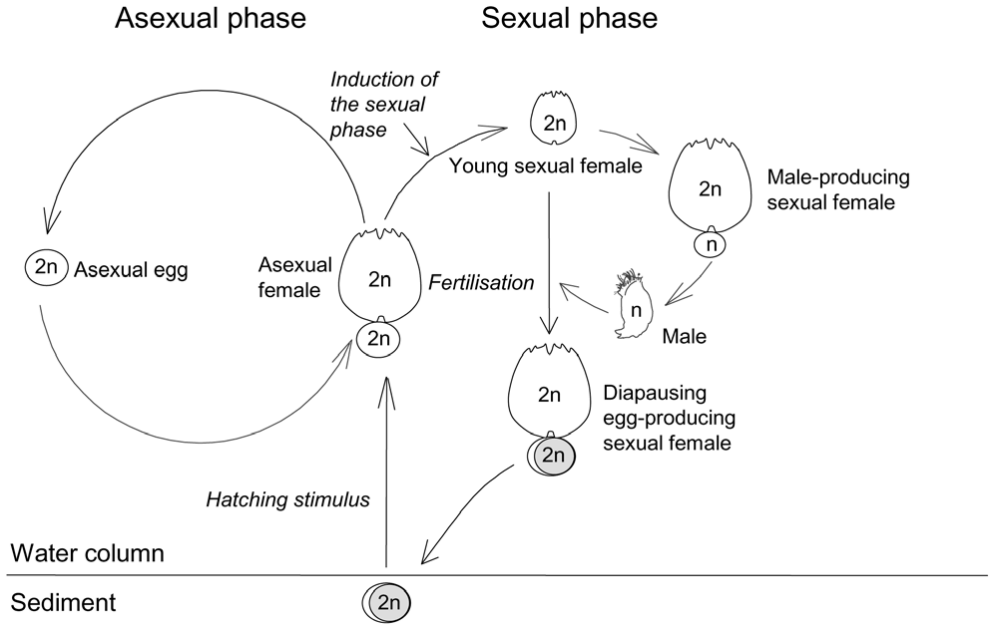
Typical life cycle of monogonont rotifers.

Particularly, the life cycle of cladocerans and monogonont rotifers is postulated to promote fast population growth and population persistence. They are cyclical parthenogens (see Fig. 1 for rotifer life cycle) that reproduce asexually over numerous generations, thereby allowing both fast colonization and a prolonged phase of clonal selection. After this period of clonal proliferation, diapausing eggs are produced by sexual reproduction which, in rotifers, is induced by environmental factors such as population density [13]. Diapausing eggs tolerate adverse conditions and a fraction of them hatches when favourable conditions resume. However, a fraction of these eggs remains viable in the sediment of ponds and lakes, forming an ‘egg bank’ that can persist for a long period of time in the habitat [14]. Hatching of diapausing eggs could imply the expression of genetic variance that has been hidden following clonal selection [15], [16]. Despite the striking life cycle evolutionary convergence between monogonot rotifers and cladocerans, some differences exist. Sexual rotifer females produce haploid eggs. If the haploid eggs are not fertilized, they develop into haploid, dwarf males. If they are fertilized, they develop into sexual, diapausing eggs. This haplodiploidy with dwarf males does not occur in cladocerans, whose males are diploid.

Genetic differentiation due to persistent founder effects would be stronger where only a few individuals found a population. In principle, a single diapausing egg has the potential to found a new population of cyclical parthenogens [17], and in fact this kind of foundation is a common practice for some experimental populations. However, the fate of a population founded by a few individuals might be strongly affected by the effects of inbreeding by drift. Inbreeding causes an increase in homozygosity, resulting in more apparent effects of deleterious alleles, since they often are recessive or partially recessive, and a loss of overdominance. [1], [2]. The increased homozygosity, in addition to epistatic effects [18], may explain the so-called ‘inbreeding depression of fitness’. Although outbreeding depression (i.e., the fitness decrease caused by breeding genetically distant individuals) should also be considered, it is clear that in a population affected by inbreeding depression by drift a new immigrant has increased opportunities to contribute to the gene pool. This would diminish the founder effect. Inbreeding depression has been detected in the cladoceran *Daphnia* [e.g.], [19,20], and evidence for the existence of mechanisms resulting in some avoidance of intraclonal crosses exist [21]. Moreover, Ebert *et al*. [22] detected hybrid vigour effects in an experimental *Daphnia* metapopulation, where immigrants were introduced in highly inbred populations. These authors observed that hybrids between residents and immigrants predominated after a growing season, suggesting that the introduction of immigrant genes into the population had been favoured.

Inbreeding depression might be transient, since homozygosity would cause deleterious recessive alleles to be exposed to purging selection. In that respect, monogonont rotifers are particularly interesting because, due to male haploidy, deleterious recessive alleles are expected to be exposed to purging selection [1], [23]. Despite this fact, to our knowledge, only a pair of old studies provides some insights into inbreeding depression in rotifers. Hertel [24] studied inbreeding depression in the rotifer *Epiphanes senta* by comparing growth rates in the parental generation and three consecutive offspring generations obtained by selfing. Evidence for hybrid vigour was also found by crossing different strains in the F_3_ generation. A later study by Birky [25] with *Asplanchna brightwelli* compared the sexual offspring of an unique clone (selfing) with the offspring resulting from crosses among clones isolated from different populations. Birky's study showed that only a low proportion of the eggs obtained by selfing hatched, and that the clones obtained from these eggs presented highly variable parthenogenetic reproduction rates and low survival. That work also provided evidence for purge of recessive detrimental alleles, which would be present in heterozygosis in the original clones, after several generations of selfing. Note, however, these two studies did not compare fitness of intraclonal and interclonal crosses within the same population.

The objective of the present work was to analyze the effects of inbreeding in the monogonont rotifer *Brachionus plicatilis* (Müller, 1786). We used an experimental design that thoroughly addressed inbreeding depression at the population level, estimated fitness along all the life-cycle steps and tested for these effects in two different populations, so that shortcomings in the previous studies for assessing inbreeding depression at population level are circumvented. *B. plicatilis* is common in brackish and saline ponds and lakes [26]. It shows high genetic differentiation among populations in the Iberian Peninsula, with clear phylogeographic structure [9], [27], [28]. The two populations tested for inbreeding depression inhabit ponds with a large difference in size, which is expected to be correlated to population size. Moreover, these two populations also differ in their investment in sexual reproduction [29]. We compared intraclonal (selfed) and interclonal (outcrossed) crosses performed in the laboratory using a factorial design, with six clones (parental clones; hereafter, P-clones) providing both males and females for the crosses. The following fitness components were measured in the offspring from those crosses (F1 generation): the F1 egg hatching proportion, the F1 clone viability (proportion of clones –founded from the hatched F1 diapausing eggs– that survived 7 days), and the initial finite growth rate *R* of the viable F1 clones. Besides these components related to the asexual reproduction, the proportion of male-producing clones and the proportion of diapausing egg-producing clones were also tested as fitness components related to sexual reproduction. Additionally to the F1 fitness components, we compared the parental fertilization proportion –i.e. the proportion of sexual females being diapausing egg-producers– between both types of crosses, in order to explore if there were signs of inbreeding avoidance. The statistical analysis tested whether the effect of the interaction between the two P-clones on the measured responses was significant. If so, it was inspected whether the interaction coefficients corresponding to selfed crosses were among the most negatives. Additionally, the existence of global differences between selfed and outcrossed crosses was tested.

An interest of this work was to know if fitness depression is expectable when a rotifer population is founded by a few individuals, given that inbreeding by drift after clonal propagation of colonizers is likely. If so, an immigrant arriving to a recently founded population would have opportunities, due to the inbreeding depression operating in such population. Although in the present experiment inbreeding depression was tested by forcing selfing, we note that the experimental set-up is equivalent to the natural situation occurring after the foundation of a new population by one or a few clones (i.e., inbreeding by drift).

## Results

**Figure 2:**
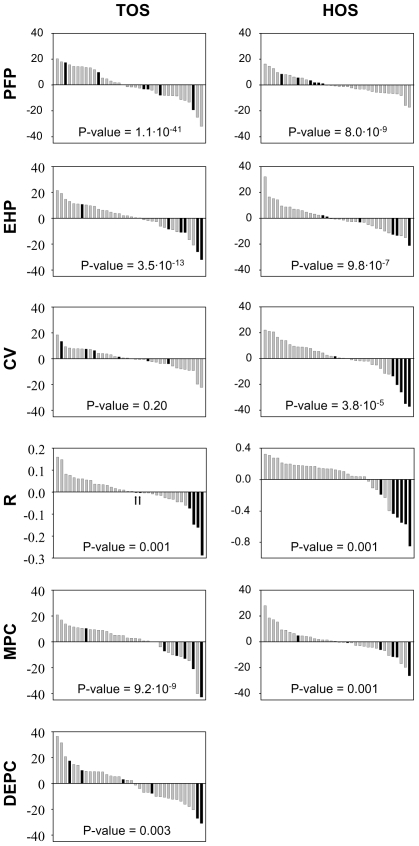
Interaction coefficient *(M×F)_ij_* for each of the 36 clonal crosses for Poza Sur (TOS) and Hondo Sur (HOS) populations. The exact P-value accounting for the statistical significance of the interaction is indicated in each graph. Black bars: selfed crosses. Grey bars: outcrossed clonal crosses. PFP: parental fertilization proportion. EHP: F1 egg hatching proportion. CV: F1 clone viability. *R* (i.e., the net growth rate of the viable F1 clones). MPC: proportion of male-producing clones. DEPC: proportion of diapausing egg-producing clones. The arrows in the graph of *R* for TOS point at two values for selfed crosses.

**Figure 3:**
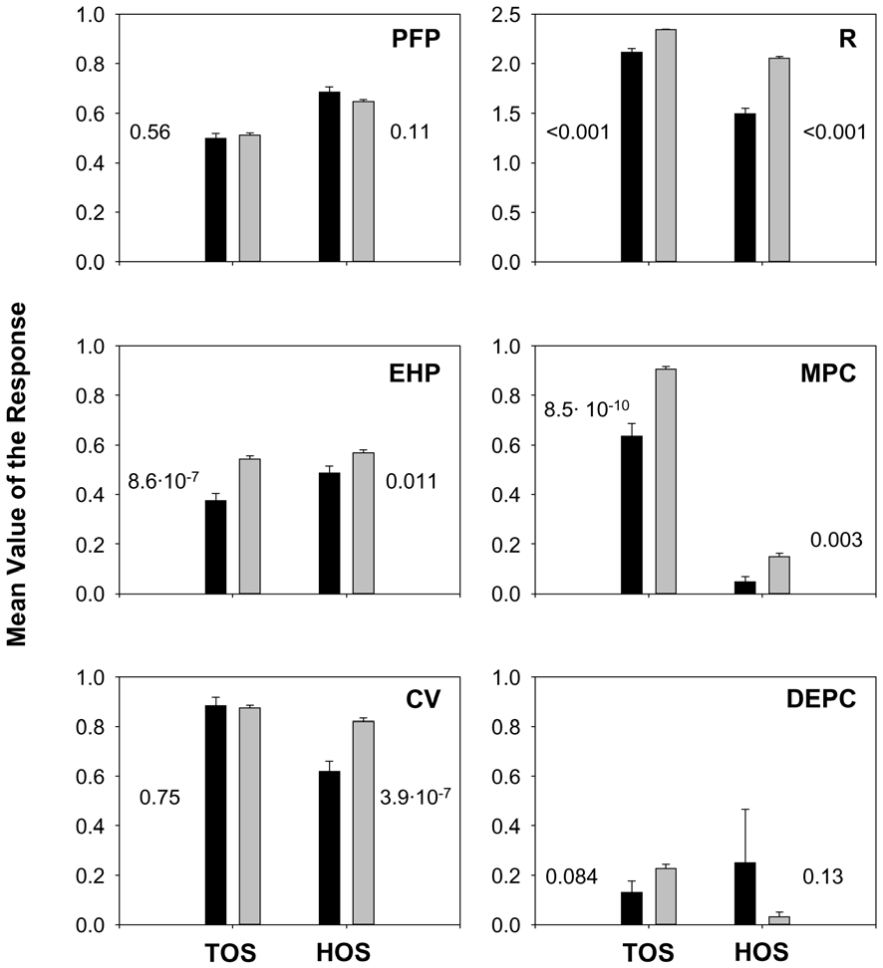
Mean (±SE) of the measured responses of selfed and outcrossed crosses for Poza Sur (TOS) and Hondo Sur (HOS) populations. P-value accounting for the significance of the effect of the type of cross is indicated in each graph. Black bars: selfed crosses. Grey bars: outcrossed crosses. Abbreviations as in Figure 2.

Parental fertilization proportions did not show a pattern of inbreeding avoidance. Although P-clone combinations had a significant interaction effect on the parental fertilization proportion, the most negative P-clone interaction coefficients did not tend to occur in selfed crosses (Fig. 2, PFP). Moreover, the global parental fertilization proportion was similar for selfed and outcrossed crosses in both TOS (Poza Sur, the smallest population; see Methods for details) and HOS (Hondo Sur) populations (Fig. 3). Similarly, evidence for inbreeding depression on the proportion of diapausing egg-producing clones was not found in any of both populations. P-clone combinations had a significant interaction effect in TOS, but selfed crosses did not showed a clear tendency to have low interaction coefficients (Fig. 2, DEPC). Moreover, in both populations a global effect of the type of cross was not observed (selfed vs. outcrossed; Fig. 3). Nevertheless, in TOS a non-significant tendency to decrease the proportion of diapausing egg-producing clones was observed in selfed crosses (Fig. 2 and 3).

Evidence for inbreeding depression was detected for the F1 generation on F1 egg hatching proportion, *R* and the proportion of male-producing clones. Fig. 2 (EHP, *R* and MPC) shows that, for both ponds, the effect of the P-clone interaction was significant for these three fitness components and, moreover, the interaction coefficients tended to be negative for the selfed crosses. Similarly, the average value of these fitness components was significantly larger for the outcrossed than for the selfed offspring, in both TOS and HOS populations (Fig. 3). The difference in hatching proportions between selfed and outcrossed F1 diapausing eggs was larger in TOS (17%) than in HOS population (8.2%). The same trend was observed for the proportion of male-producing clones. The difference between selfed and outcrossed F1 clones in the proportion of male production was 27% and 10% for TOS and HOS, respectively. In contrast, the negative effect of selfing on *R* was larger in HOS (difference between outcrossed and selfed F1 clones: 0.56) than in TOS population (difference: 0.23). Sexual reproduction in *Brachionus* is density-dependent ([30]–[32]). Consequently, the proportion of male-producing clones can be affected by the density achieved in the cultures (i.e., by *R*). A robust regression was performed to explore this relation. For each F1 clone, the log number of males produced was used instead of the associated proportion. As a positive relation was found, the regression coefficients on log *R* were used to correct the log number of males by density. Subsequently a robust one-way ANOVA showed that the density-corrected log number of males was lower in the selfed than in the outcrossed F1 clones, in both TOS and HOS (P-value<0.001 for both populations). This is consistent with the effect detected for the proportion of male-producing clones.

Results regarding the proportion of F1 clone viability varied between populations. Inbreeding depression in F1 clone viability was not observed in TOS. This fitness component was not significantly affected by the P-clone interaction, and the interaction coefficients did not tend to be negative for selfed crosses (Fig. 2, CV). Fig. 3 shows similar average values of this fitness component for the two types of offspring in TOS. In contrast, inbreeding depression was detected in HOS for this trait. Viability was significantly affected by the P-clone interaction, and the coefficients were negative for the selfed crosses with only one exception (Fig. 2). In agreement with these results, viability was 20% lower in HOS for the selfed F1 clones than for the outcrossed ones, this difference being significant (Fig. 3). It is worthy to note that, in spite of these differences in viability between the two populations, when the effects of viability and hatching proportion of the F1 diapausing eggs were combined (i.e, the F1 egg hatching proportion times the F1 clone viability), results obtained for TOS and HOS were similar. Hence, the proportion of F1 diapausing eggs which hatched and gave viable F1 clones was 33% in TOS and 30% in HOS, for selfed crosses, and 47% for both TOS and HOS, for outcrossed crosses.

## Discussion

The results of this study show that inbreeding depression affects several fitness components of *B. plicatilis* life cycle. There is clear evidence that inbreeding negatively affects fitness components associated with the asexual phase. Inbreeding depression has been found in both populations studied for the proportion of F1 diapausing eggs yielding viable clones (i.e., the F1 egg hatching proportion times the F1 clone viability), and the growth rate of the F1 clones. These findings are consistent with the negative inbreeding effects pointed out in previous studies on rotifers [24], [25]. In contrast to our findings for the asexual phase, our results for the sexual phase are less clear. Evidence for inbreeding depression in the production of males exists for both TOS and HOS populations, as shown by parameters related to male production, even when the effect of culture density on this production was discounted. Nevertheless, an inbreeding effect on the proportion of clones able to produce diapausing eggs through selfing was not found in both TOS and HOS. Lack of evidence for inbreeding depression in the proportion of clones able to produce diapausing eggs might be the result of low statistical power, since sample size decreases with the successive life-cycle steps analyzed. Alternatively, most of the deleterious effects could be expressed in the embryo developmental process of the diapausing eggs and in the parthenogenetic proliferation of the first generations, because these phases involve many gene functions. Thus, our demonstration of inbreeding depression represents the first thorough study measuring fitness step by step throughout the life cycle in the phylum Rotifera.

Concerning cladocerans, another group of cyclical parthenogenetic zooplankters, previous studies of several species belonging to the genus *Daphnia* revealed that inbreeding depression affects life-cycle fitness components [19], [20], [33]–[35], although there is no consistent evidence for an inbreeding effect on sexual phase [35], [36]. Therefore, there are some qualitative similarities about cyclical parthenogenetic continental zooplankton. However, evidence for the existence of a mechanism to diminish the probability of selfing has been observed in *Daphnia* populations [21], which consist of an uncoupling of male and sexual female production within the same clone. Rotifer life cycle does not allow such uncoupling, because the fate of a sexual rotifer female (being either male or diapausing-egg producer) is controlled by her copulation when young and thus by the density of males in the population. On the other hand, our results suggest lack of selfing avoidance in *B. plicatilis*, since the parental fertilization proportion of selfed and outcrossed crosses did not differ. In fact, the results of the reproductive isolation experiments performed by Gómez and Serra [37] and Suatoni et al. [38] with the *B. plicatilis* species complex do not reveal a pattern of male discrimination between females belonging to the same and to a different clone within the same species.

In contrast with the results in cladocerans, the inbreeding depression detected in our study might be unexpected because rotifer males are haploid, so they are expected to purge deleterious recessive mutations [39]. Studies with other haplodiploid taxa –mainly insects- have shown both results for [23], [40] and against [41], [42] the existence of inbreeding depression. In these groups, the general trend seems to show inbreeding depression in haplodiploid taxa, but with lower intensity than in the case of diploids [reviewed in 40]. There may be different explanations for ineffective purging in haplodiploid rotifers. First, rotifer males are functionally simple and live short lives when compared to females [43]. Thus, they would be expected not to express a large proportion of their genes; such genes not being exposed to natural selection. In this sense, it is worthy to note that most fitness components measured here do not occur in males, although these components certainly should imply fundamental physiological processes. Second, even in the case of traits showed by both sexes, deleterious alleles could be maintained by several mechanisms. Hence, rotifer males might be expressing the maternal genotype, as suggested by Birky [25]. In addition, Henter [40] suggested other possibilities, such as (1) a different genetic control for the trait for both sexes, (2) that the fitness reduction was due to the loss of overdominant heterozygotes under inbreeding or (3) a weak selection for the trait in the field. The last possibility seems unlikely for the case studied here, given the type and the intensity of the effects of inbreeding that were found. Third, the observed inbreeding depression could be the resulting equilibrium between purging in the haploid males and a high deleterious mutation accumulation during the asexual generations.

If inbreeding depression is not due to an advantage of the heterozygote due to overdominance [40], populations subject to regular inbreeding would be expected to purge their genetic load, because the level of homozygosis would increase, and recessive deleterious genes would be exposed to natural selection [1], [40], [44], [45]. Again, the inbreeding depression detected here reveals that such a mechanism has not operated in our rotifer populations: inbreeding is not a recurrent feature in rotifer populations. In well-established rotifer natural populations, inbreeding due to non-random mating is extremely rare, as suggested by Gómez *et al* [9], after their finding of Hardy-Weinberg equilibrium for microsatellites in the rotifer populations studied here, which has been confirmed by Campillo *et al*. (unpublished data) by using a higher number of microsatellites. Moreover, purging efficiency may be limited, even when there is a certain level of inbreeding in the population, as this efficiency needs the average effect of deleterious mutations to be strong relative to the effective population size [2], [46]. Most rotifer populations are of large size, even when populations go through a period of diapause. For instance, average density of viable diapausing eggs in the upper sediment (0–2 cm) of TOS is around 20 eggs/cm^3^ [47], which gives huge abundances even in a small pond. The high abundances of rotifer populations would hinder an efficient purge of recessive deleterious alleles through inbreeding by non-random mating and, furthermore, through inbreeding by drift, being this type of inbreeding highly unlikely in view of such abundances. The similar results found for TOS and HOS populations are surprising, because differences in inbreeding depression could be expected due to differences in habitat size (if correlated to population size) and sex investment. If compared to TOS, HOS is a population inhabiting a larger pond, tending to show lower investment in sex and lower density of diapausing eggs in the sediment [29]. Fitness reduction in inbred *Daphnia* has been found to be negatively related to levels of investment in sex [48]. On the other hand, inbreeding depression can be expected to be lower in small populations, where purging would have been more effective. According to this, inbreeding depression would be expected to be lower in TOS than in HOS. A possible explanation for our results would be that in HOS low sex investment could result in bottlenecks during the dormant phase of the population, which would imply lower effective population sizes and a more effective deleterious mutation purging.

Inbreeding by drift would not be important in well-established rotifer populations due to their typically high population sizes and clonal diversity [9]. However, it could occur in the colonization period of a new habitat if the founding event involves few individuals, as stated by the Monopolization Hypothesis. There is genetic evidence supporting that rotifer and cladoceran populations are founded by few clones [9], [49], [50]. If so, the numerical advantage of the first clone to arrive would be partially diminished by inbreeding depression after sexual reproduction, due to the higher fitness of the offspring between the first colonizer and subsequent immigrants in comparison with selfed offspring from the first colonizer. Louette *et al*. [49] observed that the frequency of initially rare alleles increased in newly created *Daphnia* populations during the first three years. In a similar study by Ortells *et al*. (unpublished data) a new low frequency microsatellite allele was detected in the second growing season (i.e., after the first sexual reproduction episode, when the effects of inbreeding are expected to appear). Furthermore, recolonization experiments in a metapopulation of cladocerans, using several ponds with highly inbred populations that were supposed to be locally adapted, have shown hybrid vigour effects [22], and Haag *et al*. [50] found that this hybrid vigor seemed to influence temporal changes in population genetic diversity. Contrasting with these findings, in their study on the rotifer *B. plicatilis* in Eastern Spain, Gómez *et al*. [9] found fixation index (Fst) values consistent with a single clone as founder of these rotifer populations. A possible explanation is that inbreeding depression would be purged after foundation and before new immigrations, which unexpectedly implies that the rate of immigration is relatively low.

In conclusion, the present study shows that rotifers can be affected by inbreeding depression. These results provide data for a phenomenon poorly studied in rotifers if compared to other taxa of similar ecological genetics (e.g., cladocerans). The results suggest lack of an efficient purge of recessive deleterious genes in already established rotifer populations, despite this organisms being haplodiploids. The inbreeding depression detected here is important when a rotifer population is founded and selfing is likely to occur. Thus, our results prompt questions on the processes occurring during the foundation of new populations that could affect gene flow between populations, and consequently their genetic differentiation. Further studies on purging efficiency during such foundations, and research on the effects of interpopulation hybridization between well-established rotifer populations –i.e., studies on outbreeding depression- would shed more light on these topics.

## Methods

### Parental Clones

P-clones for the cross-mating experiments were founded from hatchlings of *B. plicatilis* diapausing eggs obtained from the superficial sediments of two coastal ponds located in Eastern Spain: Poza Sur de Torreblanca (TOS), in “Prat de Cabanes-Torreblanca” Nature Reserve, and Hondo Sur (HOS), in “El Hondo de Elche” Nature Reserve. *B. plicatilis* diapausing egg banks had been previously detected in these ponds [27], [51], [52]. TOS is a small seasonal pond of about 0.01 Km^2^ [53], whereas HOS is semi-permanent and has an average area of 0.20 Km^2^ [54].

Diapausing eggs morphologically identified as putative *B. plicatilis* eggs were isolated from the sediment samples by the sugar flotation technique described in Gómez & Carvalho [51] with small modifications [52]. Isolated eggs were transferred individually into 96-multiwell dishes (Nunc™) containing 150 µl of 6 g L^−1^ artificial seawater (Instant Ocean®, Aquarium Systems) and incubated at 25°C under constant illumination (150–170 µmol quanta m^2^ s^−1^) for hatching [52], [55], [56]. The wells were checked daily for hatchlings up to the fourteenth day. The medium was renewed every other day to prevent fungal and bacterial growth [52]. 50 µl of culture medium were added to each well where a newborn female was observed. The culture medium was 12 g L^−1^ modified f/2 medium [57] prepared with artificial seawater (Instant Ocean®, Aquarium Systems), and contained approximately 500,000 live cells mL^−1^ of the microalgae *Tetraselmis suecica*. After a few days of clonal propagation from the diapausing egg hatchlings, the rotifer cultures were transferred to a larger culture volume, and maintained under constant illumination (approximately 35 µmol quanta m^2^ s^−1^) and at 18°C or 20°C for the HOS and TOS clones, respectively. These conditions were used for the maintenance of the clones and cross-mating experiments, and were selected because preliminary experiments showed their suitability for obtaining high fertilization proportions in crosses. Due to the fact that *B. plicatilis* belongs to a cryptic species complex [38], [56], after screening the clones according to their morphological features [58], species identification was performed using restriction analysis on a cytochrome oxidase subunit I mitochondrial gene fragment [59]. From the clones identified as *B. plicatilis*, those with both high population growth rates and high levels of sexual reproduction were selected as P-clones. The relatedness of these clones was not checked. However, they were founded from diapausing (i.e., sexual) egg banks in Hardy-Weinberg equilibrium [9], so that they are different clones from a panmictic population. Interestingly, *B. plicatilis* TOS and HOS populations harbour high genetic diversity [9]

### Cross-Mating Experiments

Using six P-clones, clonal crosses were made by combining males and females of each clone, so that 36 combinations (6 selfed and 30 outcrossed) were carried out for each local population (pond). An outcrossed clonal cross is one where the male-supplier clone (male P-clone) and the female-supplier clone (female P-clone) are different. We call selfed crosses to the intraclonal crosses, because intraclonal mating in rotifers is genetically equivalent to selfing in hermaphrodite organisms. All experiments took place between clones isolated in the same pond.

Males and females used in the crosses were produced by setting up a 100 mL culture for each P-clone. The density of females was determined daily for each culture and, when a value of at least 100 individuals/mL^−1^ was reached, approximately a quarter of the culture, including rotifers, was replaced daily with fresh culture medium. This procedure tended to generate a constant high population density at the time of daily dilution, and yielded high numbers of males and sexual females, whose production increases with density [30], [60], [61].

Virgin, newborn individuals of both sexes were used for the crosses. This was required to control paternity, but also because, (1) male fertilization ability declines after 8 hours of age [62], (2) males select young females [63], and (3) haploid egg fertilization declines in females older than 4 hours of age [62]. From the P-clones, aliquots of approximately 10 ml of the rotifer cultures were gently shaken in order to detach the eggs carried by the mothers [63]. The detached eggs exhibiting embryo movement (i.e., in an advanced stage of development) were picked out. These eggs usually hatch in less than 4–5 hours. Eggs with male embryos can be easily distinguished from eggs with female embryos due to the smaller size of the former. One hundred eggs with male embryos from the male P-clone and one hundred eggs with female embryos from the female P-clone were transferred to 1 mL of culture medium in a well of a multiwell plate (Iwaki brand, Asahi Techno Glass). This small volume facilitates male-female encounters, but individuals can swim freely, given their small size [58]. After allowing 24 hours for mating, females, which at this point could not be differentiated as sexual or asexual, were isolated individually in wells of a multiwell plate (Nunc™) with 150 µl of culture medium. After 2–3 days, females were classified according to the type of egg and offspring they produced as: (1) asexual females (those producing female offspring), (2) unfertilized sexual females (those producing male offspring), or (3) fertilized sexual females (those producing diapausing eggs). Some of the latter females produced several eggs. From these data we calculated the parental fertilization proportion as the proportion of sexual females producing diapausing eggs. In order to obtain at least 30 diapausing eggs from each of the 36 clonal crosses for each population, this procedure was repeated up to a maximum of four times per clonal cross.

The diapausing eggs obtained were stored in darkness at 4°C [52] in 60 g L^−1^ artificial seawater to allow them to complete their obligate period of diapause [64]. In this work, these eggs are called F1 diapausing eggs and the clones produced by asexual proliferation after these eggs hatched, F1 clones. Each F1 clone was grown individually. F1 diapausing eggs and F1 clones are designated as selfed or outcrossed depending on the type of cross that originated them.

### Food Preparation for Hatching of F1 Diapausing Eggs and Culture of F1 Clones

F1 clones were fed with frozen microalgae (*T. suecica*). This procedure allowed us to use microalgae from a single algal stock throughout the experiment, thereby minimizing food quality variation [29]. In order to obtain the frozen microalgae, we cultivated 64 L of *T. suecica* under the conditions described in the “Parental clones” section. When the density achieved was sufficient to supply the food necessary for the whole experiment, algae were concentrated by centrifugation at 3000 rpm for 5 minutes. We estimated the resulting density by counting under an optical microscope, using a Fuchs-Rosenthal chamber. After centrifugation, we adjusted the algal density to 15×10^6^ cells mL^−1^. This algae medium was distributed in Eppendorf® vials which were frozen at –80°C until required, when they were defrosted at room temperature.

### Hatching Induction of the F1 Diapausing Eggs

After a minimum of three months in diapause, hatching of F1 diapausing eggs was induced. A single egg from each mother was used. Hatching induction conditions were: 25°C temperature, constant illumination (150–170 µmol quanta m^−2^ s^−1^), 12 g L^−1^ salinity, and 500,000 *T. suecica* cells mL^−1^. Wells were checked for hatchlings every 12 hours for 3 weeks, and the medium was renewed once a week. The F1 egg hatching proportion was computed for each clonal cross. An egg was considered hatched when the hatchling had completely emerged from the egg and was able to swim freely.

### Measurement of F1 Clone Fitness Components

Females hatched from F1 diapausing eggs were isolated individually in 30 ml of culture medium and allowed to reproduce for 7 days. On day 7, the culture was fixed by adding 150 µl of a Lugol's solution to facilitate counting. In the preserved samples, the number of females, males and diapausing eggs produced during the clonal culture were counted. From these data, the following parameters were calculated: (1) F1 clone viability. An individual F1 clone was considered viable if at least one female was alive on the seventh day of culture. For each clonal cross, F1 clone viability was computed as the proportion of the hatched F1 diapausing eggs resulting in viable clones in culture. (2) The initial net growth rate (*R*) was calculated for each viable F1 clone using the equation N*_t_ = *N*_0_ R^t^*, where N*_t_* is the number of females on the seventh day of culture (*t* = 7), and N*_0_* = 1. Note that several *R* values were estimated for each clonal cross. (3) The proportion of male-producing clones was calculated for each clonal cross as the proportion of clones where males were observed among the viable F1 clones. (4) The proportion of diapausing egg-producing clones was calculated for each clonal cross as the proportion of male-producing F1 clones that produced diapausing eggs (the result of successful selfing within the F1 clone), so taking into account that diapausing egg production requires the previous production of males.

### Data Analysis

Measurements performed for both the clonal crosses and the F1 generation resulted in estimations of six response parameters: the parental fertilization proportion, the F1 egg hatching proportion, the F1 clone viability, the initial net growth rate R, the proportion of male-producing clones and the proportion of diapausing egg-producing clones. According to the experimental design, a response parameter can be described using the linear model:
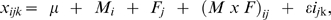
where *x_ijk_* is the value of the response (e.g., *R*) measured in the *k*-th replicate of the cross between the *i*-th male P-clone and the *j*-th female P-clone, *μ* is the average value of *x_ijk_* under experimental conditions, *M_i_* is the male P-clone effect, *F_j_* is the female P-clone effect, *(M×F)_ij_* is the effect of the interaction between the two P-clones, and *ε_ijk_* is an error term. For five of the six responses, *x_ijk_* is a binary variable; for example, a F1 diapausing egg hatches (*x_ijk_* = 1) or not (*x_ijk_* = 0), and each individual case acts as a replicate. The exception is *R*, for which *x_ijk_* is a real, non-negative variable. The linear model allows us to estimate if there are significant effects of the male P-clone, of the female P-clone or of the interaction between the two P-clones on a given response parameter. Using the model, inbreeding depression was analyzed for each of the response parameters and population by assessing if the interaction effect was significant. Where the interaction was significant, the 36 coefficients accounting for the interaction, *(M×F)_ij_*, were inspected to test if the *(M×F)_ii_* (selfed crosses) tended to be lower than *(M×F)_ i j≠i_* (outcrossed crosses). Statistical significance of the effects was analyzed by means of a robust ANOVA (a two factors generalization of the Welch test) [65] in the case of *R*; for the remaining parameters generalized linear models for binary data assuming a Binomial distribution for data and logit as link function were used.

Additionally, global differences between selfed and outcrossed crosses or offspring were tested. For *R*, a robust *a priori* planned mean contrast was performed, this analysis using a generalization of the Yuen method [65]. For the rest of the responses measured, generalized linear models for binary data assuming a Binomial distribution for data and logit as link function were used. In all cases, two-tail tests were used.

As indicated above, sexual reproduction in *Brachionus* depends on population density. As a consequence, levels of sexual reproduction in F1 might be correlated to *R*, since *R* is indicative of the density achieved in the F1 clone cultures. In order to correct a putative effect of culture density on the levels of sexual reproduction, the following approach was used: (1) Robust regressions [65], [66] between log *R* and the log number of males produced per F1 clone or the log number of diapausing eggs per F1 clone were performed for each population and type of cross (selfed and outcrossed). Before log transformation, we added 1 to the dependent variables, since in some cases their value was zero. Note that male and diapausing egg production are related but different to the proportion of male-producing clones and the proportion of diapausing egg-producing clones, respectively, and were used to fit the requirements of a regression. The regressions yielded non-significant results for the case of diapausing eggs produced per F1 clone. (2) The regression coefficients were used to correct the log number of males produced per F1 clone by the density, since these two variables were found positively related in most of the population x type of cross combinations. After this correction, a robust one-way ANOVA (a robust generalization of the Welch test) [65], [66] with the type of cross as factor of classification was performed.

All the statistical analyses were carried out using R statistical software v. 2.7.2 (The R Foundation for Statistical Computing, 2008), using functions included in the WRS package [67] in the case of the robust tests.
